# Supplementary material for: Mapping kinase domain resistance mechanisms for the MET receptor tyrosine kinase via deep mutational scanning
Source: bioRxiv. 2024 Dec 5:2024.07.16.603579. Originally published 2024 Jul 18. Preprint. [Version 2] doi: 10.1101/2024.07.16.603579 (PMC11275805; doi:10.1101/2024.07.16.603579)
Supplement: Supplement 2 [file NIHPP2024.07.16.603579v2-supplement-2.pdf]

bioRxiv preprint doi: https://doi.org/10.1101/2024.07.16.603579; this version posted December 5, 2024. The copyright holder for this preprint (which was not certified by peer review) is the author/funder, who has granted bioRxiv a license to display the preprint in perpetuity. It is made available under aCC-BY 4.0 International license.

Supplemental information

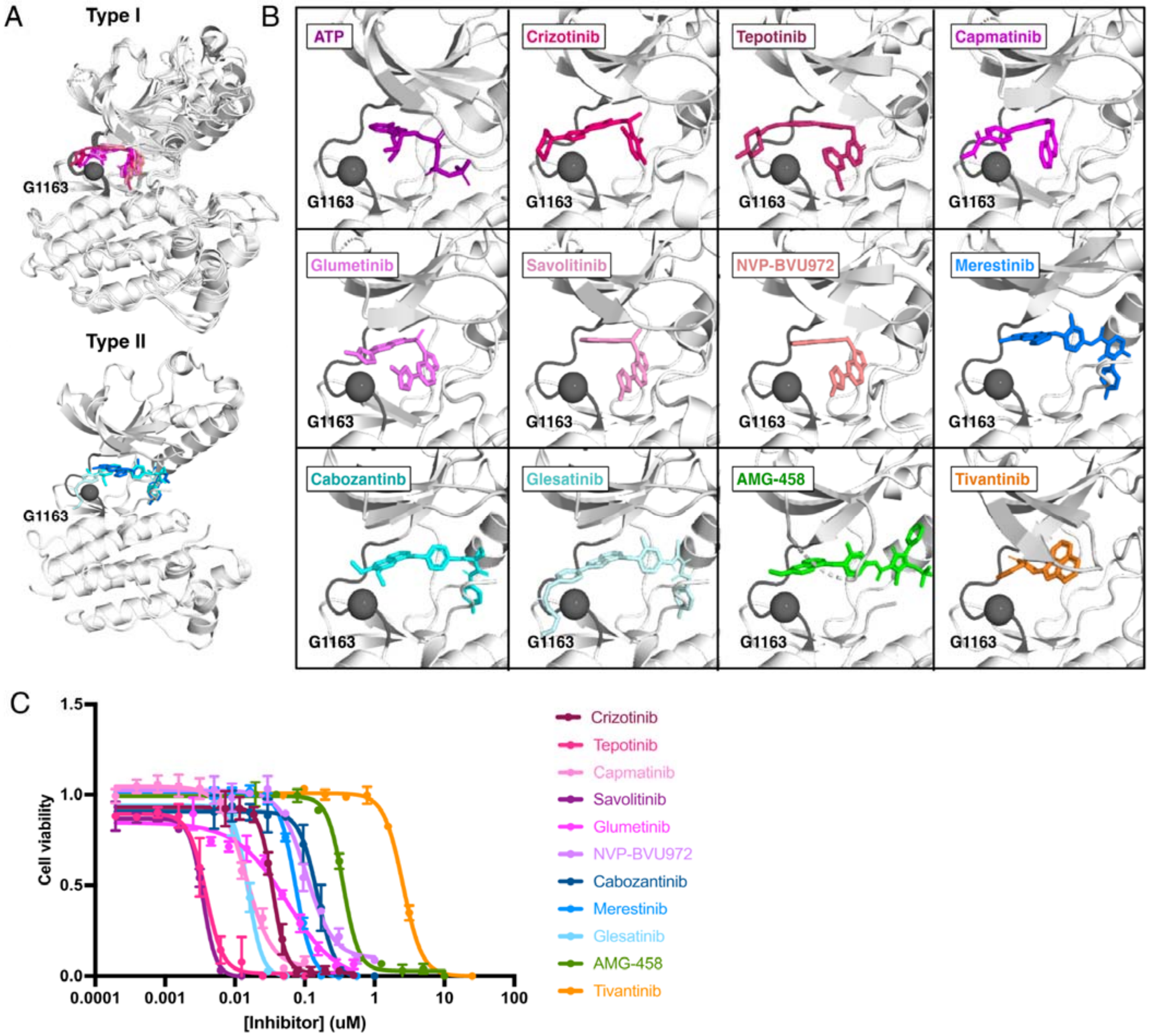

bioRxiv preprint doi: https://doi.org/10.1101/2024.07.16.603579; this version posted December 5, 2024. The copyright holder for this preprint (which was not certified by peer review) is the author/funder, who has granted bioRxiv a license to display the preprint in perpetuity. It is made available under aCC-BY 4.0 International license.

Figure 1 - figure supplement 1. Structural inhibitor classification and dose-response determination. (A) Type I (crizotinib, 2WGJ; tepotinib, 4R1V; capmatinib; savolitinib, 6SDE; NVP-BVU972, 3QTI) and type II (merestinib, 4EEV; cabozantinib; glesatinib) inhibitor-bound MET kinase domain structures globally aligned. Hinge (gray) and G1163 (represented as a sphere) are highlighted to show the kinase domain solvent-front relative to each inhibitor. Inhibitors lacking experimental structures (capmatinib, cabozantinib, glumetinib, and glesatinib) were docked onto a representative type I (PDB 2WGJ) and type II (4EEV) structure through AutoDock Vina **(Eberheart et al., 2021; Trott et al., 2010)**. (B) Solvent-front and G1163 highlighted relative to the ATP-bound kinase domain crystal structure (3DKC) and all inhibitors screened. (C) Dose-response curves for each inhibitor against the TPR-fusion MET and METΔEx14 intracellular domains, stably expressed in Ba/F3 cells.

bioRxiv preprint doi: https://doi.org/10.1101/2024.07.16.603579; this version posted December 5, 2024. The copyright holder for this preprint (which was not certified by peer review) is the author/funder, who has granted bioRxiv a license to display the preprint in perpetuity. It is made available under aCC-BY 4.0 International license.

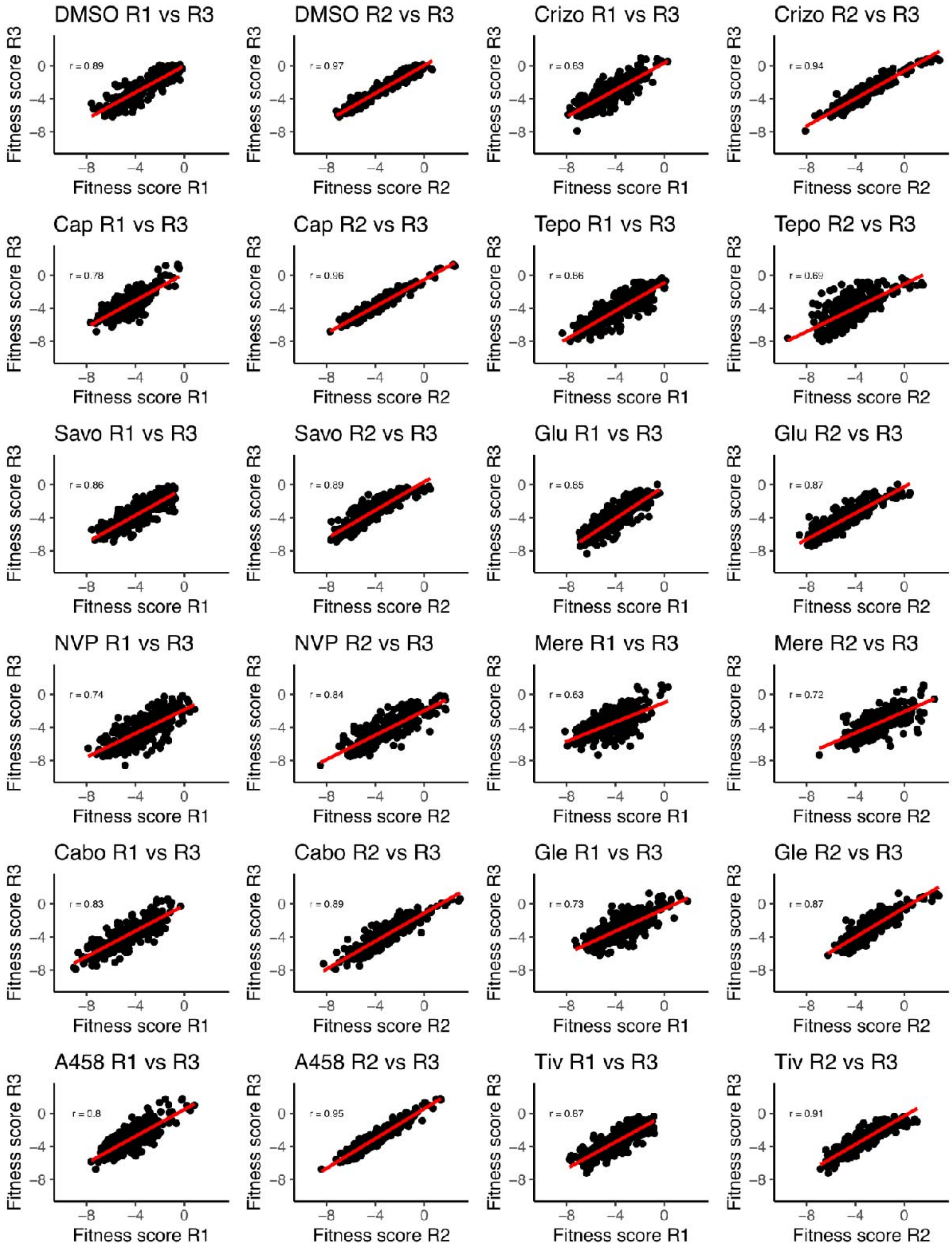

Figure 1 - figure supplement 2. Correlation analysis of the MET kinase domain site saturation mutagenesis library across replicates and conditions. Replicate correlation analysis for each inhibitor for both the TPR-fusion MET background scores with Enrich2.

bioRxiv preprint doi: https://doi.org/10.1101/2024.07.16.603579; this version posted December 5, 2024. The copyright holder for this preprint (which was not certified by peer review) is the author/funder, who has granted bioRxiv a license to display the preprint in perpetuity. It is made available under aCC-BY 4.0 International license.

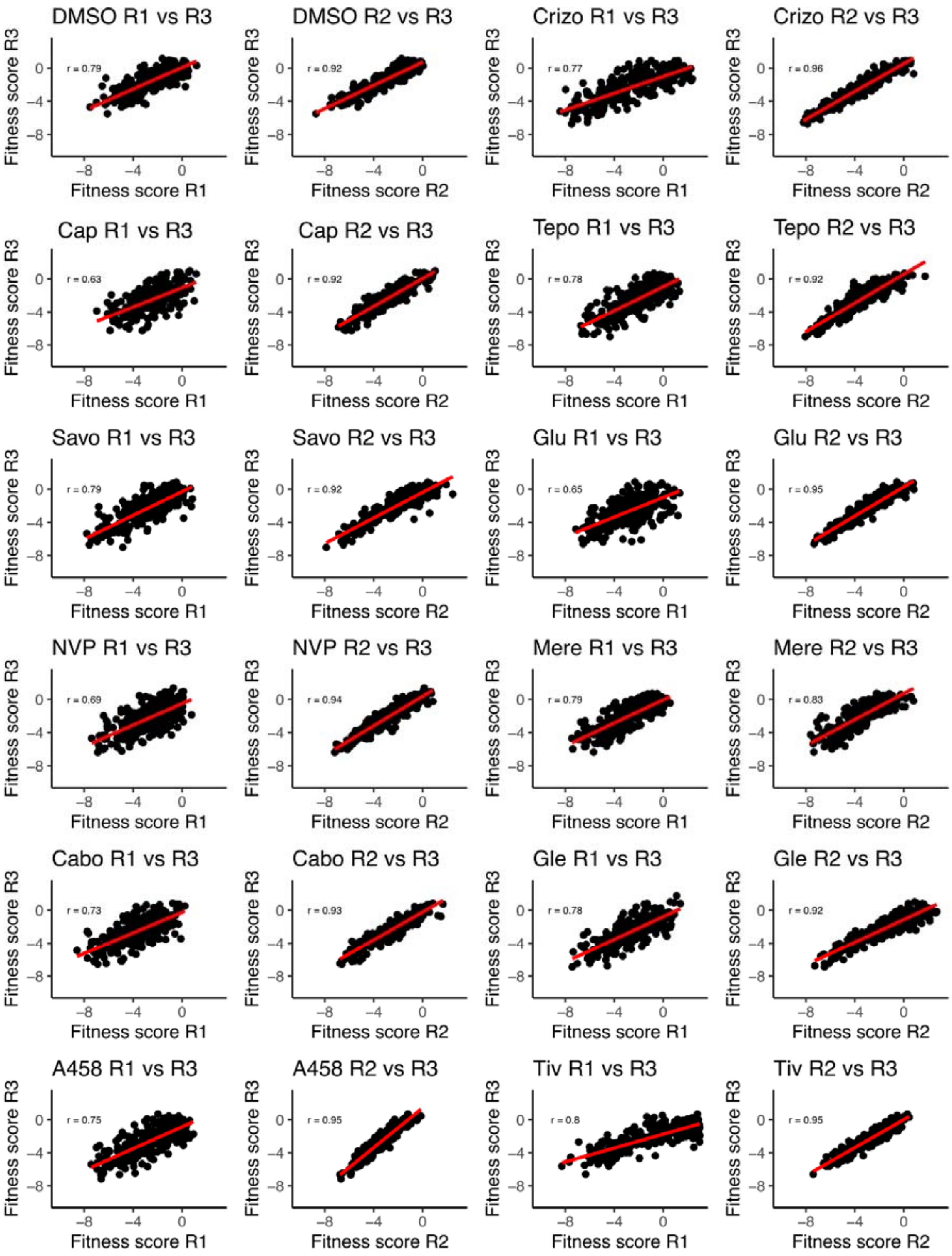

bioRxiv preprint doi: https://doi.org/10.1101/2024.07.16.603579; this version posted December 5, 2024. The copyright holder for this preprint (which was not certified by peer review) is the author/funder, who has granted bioRxiv a license to display the preprint in perpetuity. It is made available under aCC-BY 4.0 International license.

Figure 1 - figure supplement 3. Correlation analysis of the METΔEx14 kinase domain site saturation mutagenesis library across replicates and conditions. Replicate correlation analysis for each inhibitor for the TPR-fusion METΔEx14 background score with Enrich2.

bioRxiv preprint doi: https://doi.org/10.1101/2024.07.16.603579; this version posted December 5, 2024. The copyright holder for this preprint (which was not certified by peer review) is the author/funder, who has granted bioRxiv a license to display the preprint in perpetuity. It is made available under aCC-BY 4.0 International license.

MET kinase domain heatmaps (1/4)

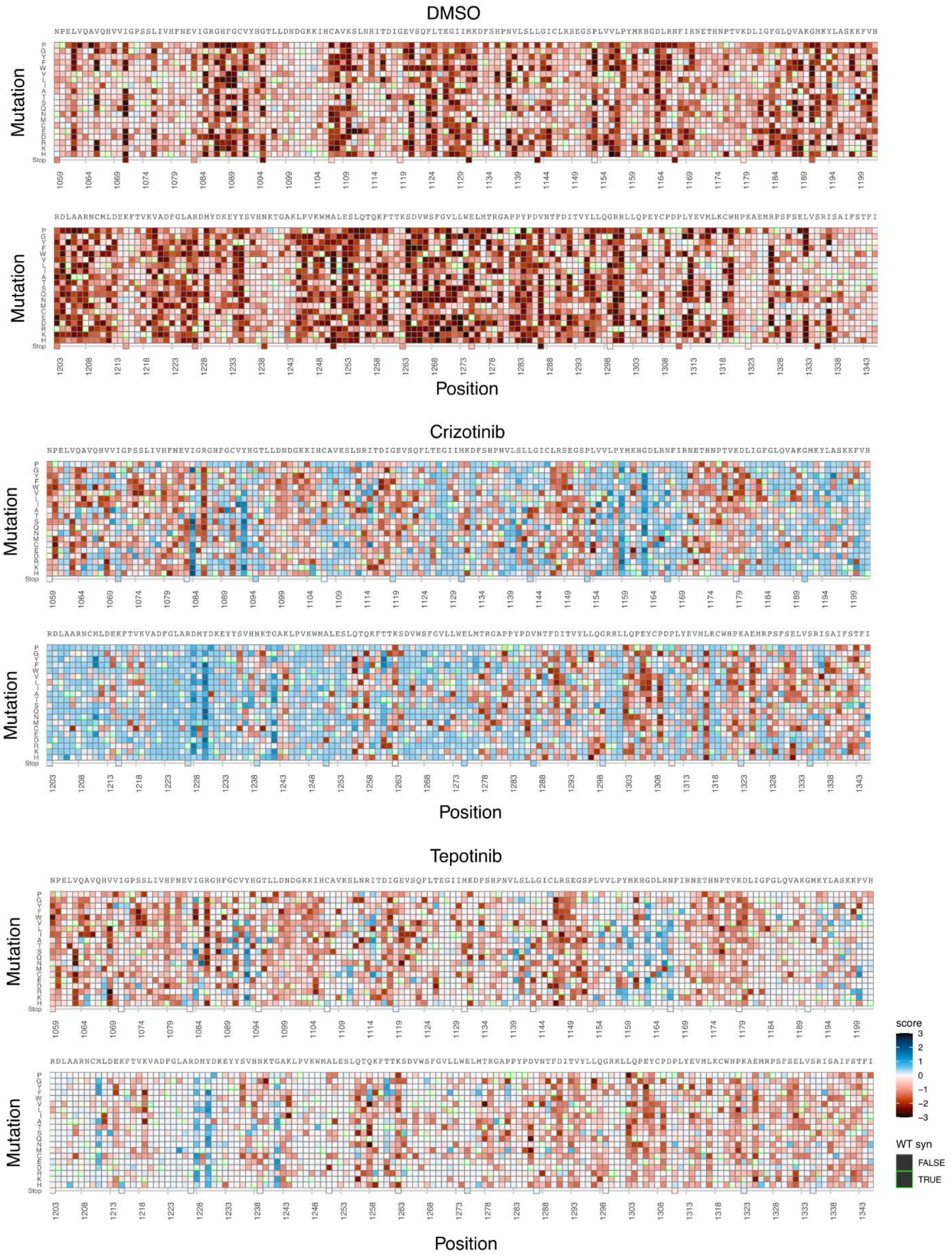

bioRxiv preprint doi: https://doi.org/10.1101/2024.07.16.603579; this version posted December 5, 2024. The copyright holder for this preprint (which was not certified by peer review) is the author/funder, who has granted bioRxiv a license to display the preprint in perpetuity. It is made available under aCC-BY 4.0 International license.

MET kinase domain heatmaps (2/4)

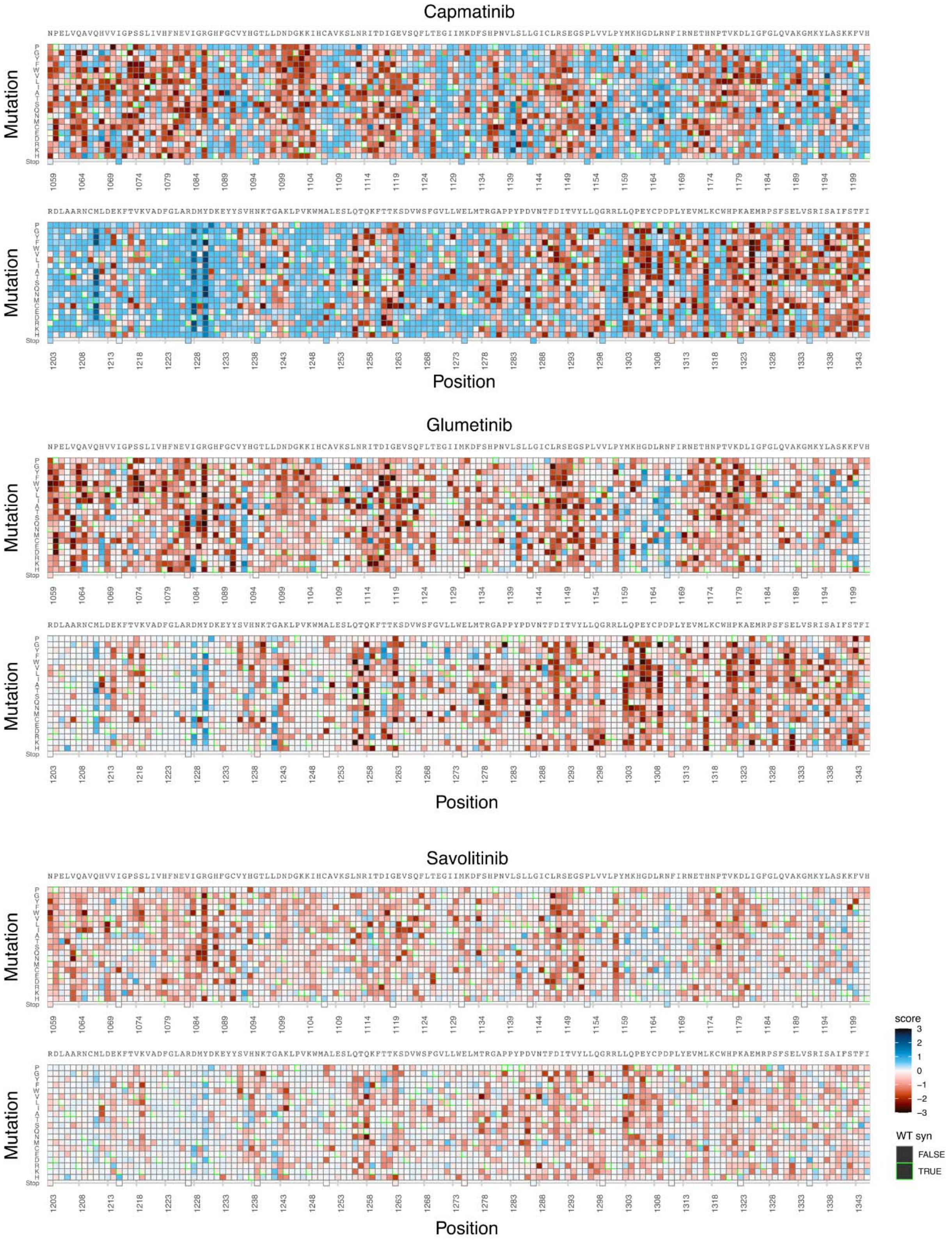

bioRxiv preprint doi: https://doi.org/10.1101/2024.07.16.603579; this version posted December 5, 2024. The copyright holder for this preprint (which was not certified by peer review) is the author/funder, who has granted bioRxiv a license to display the preprint in perpetuity. It is made available under aCC-BY 4.0 International license.

MET kinase domain heatmaps (3/4)

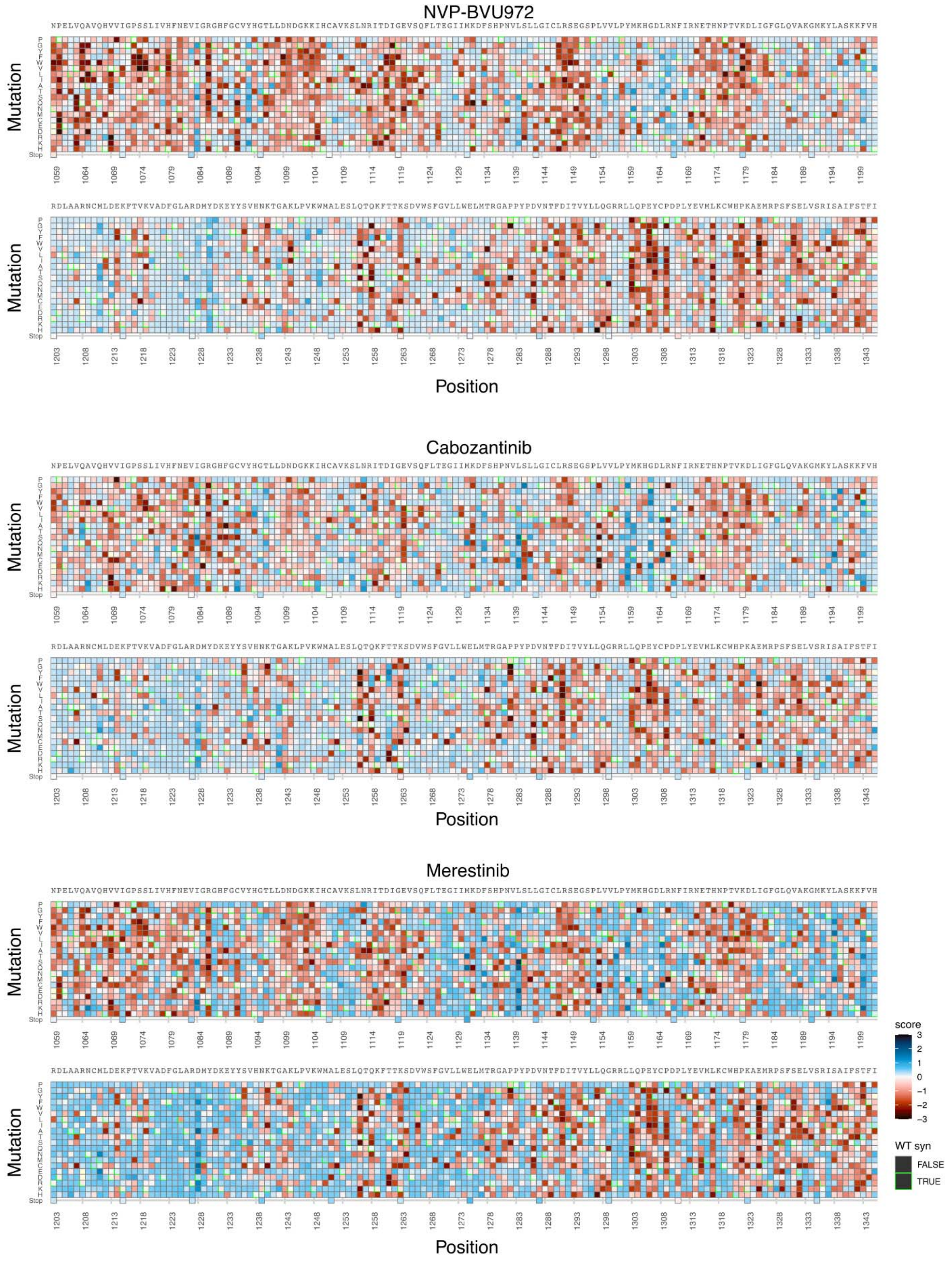

bioRxiv preprint doi: https://doi.org/10.1101/2024.07.16.603579; this version posted December 5, 2024. The copyright holder for this preprint (which was not certified by peer review) is the author/funder, who has granted bioRxiv a license to display the preprint in perpetuity. It is made available under aCC-BY 4.0 International license.

MET kinase domain heatmaps (4/4)

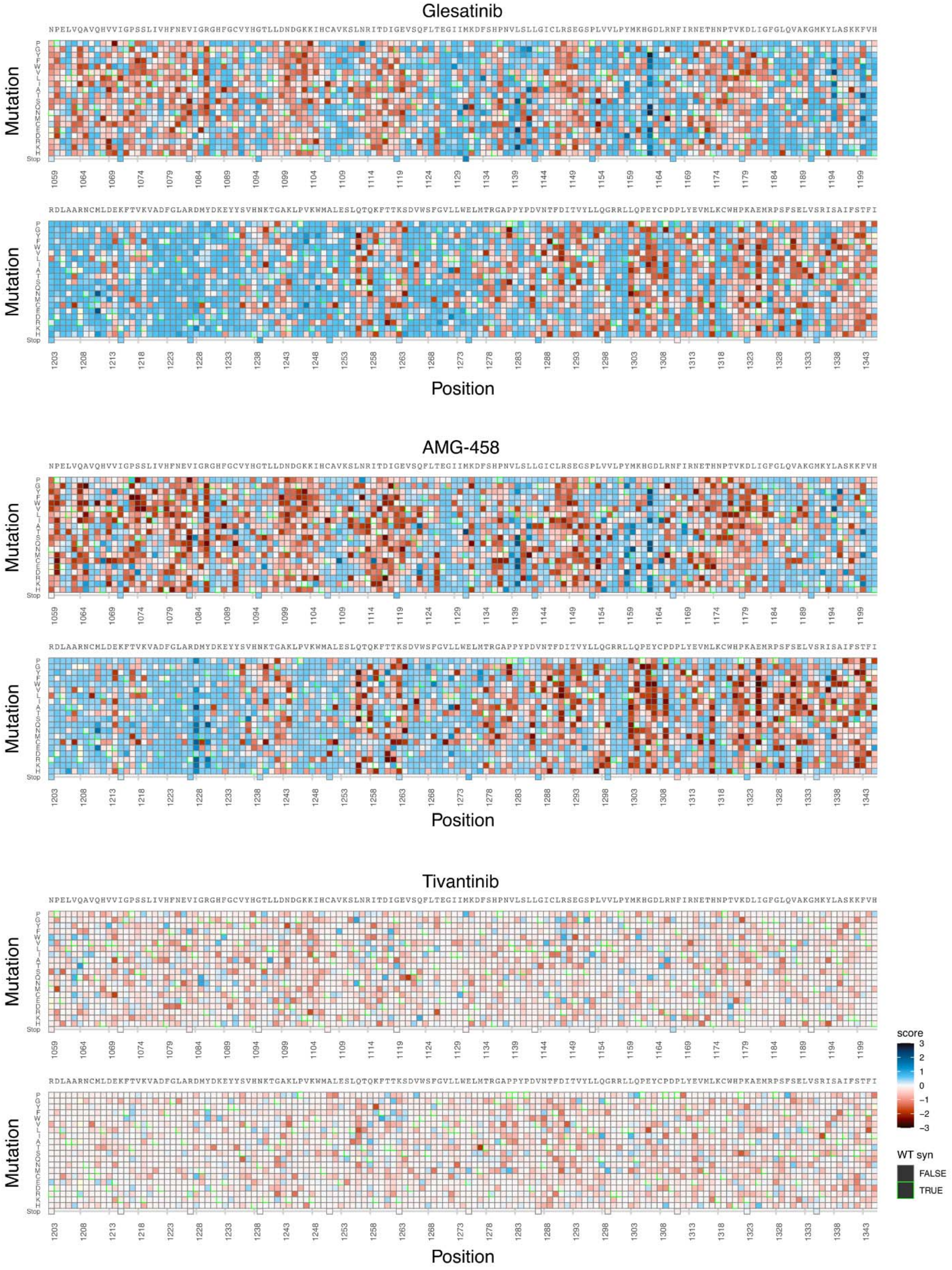

bioRxiv preprint doi: https://doi.org/10.1101/2024.07.16.603579; this version posted December 5, 2024. The copyright holder for this preprint (which was not certified by peer review) is the author/funder, who has granted bioRxiv a license to display the preprint in perpetuity. It is made available under aCC-BY 4.0 International license.

Figure 1 - figure supplement 4 . Fitness landscapes of the MET kinase domain against a panel of 11 inhibitors. Heatmap for the DMSO control condition and all inhibitor fitness scores from Rosace, subtracted from DMSO for >99% of MET kinase domain variants in the full intracellular domain background in the context of the TPR-fusion. Wild-type synonymous mutations are highlighted in green, and mutations that were not captured by the screen are in light yellow.

bioRxiv preprint doi: https://doi.org/10.1101/2024.07.16.603579; this version posted December 5, 2024. The copyright holder for this preprint (which was not certified by peer review) is the author/funder, who has granted bioRxiv a license to display the preprint in perpetuity. It is made available under aCC-BY 4.0 International license.

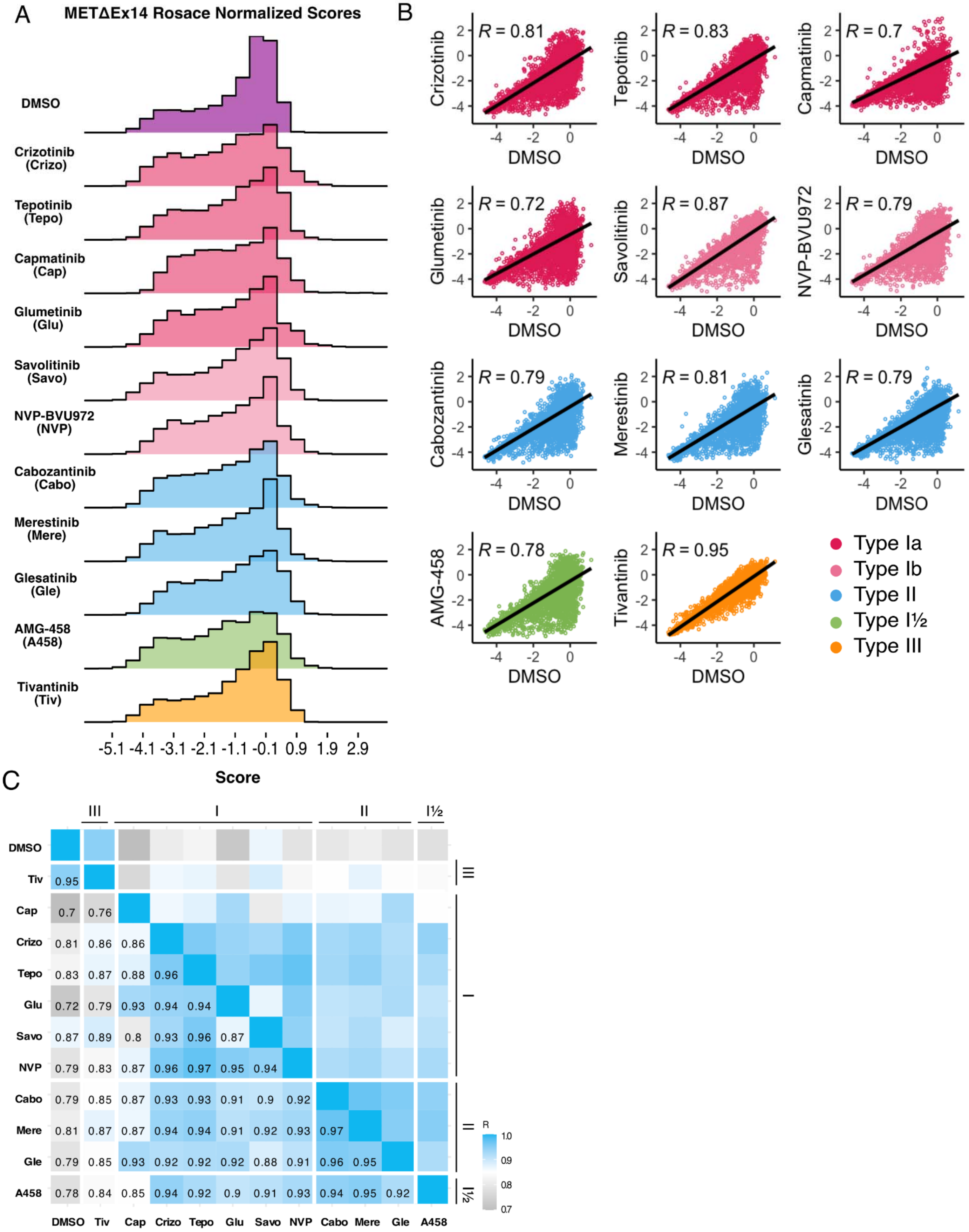

bioRxiv preprint doi: https://doi.org/10.1101/2024.07.16.603579; this version posted December 5, 2024. The copyright holder for this preprint (which was not certified by peer review) is the author/funder, who has granted bioRxiv a license to display the preprint in perpetuity. It is made available under aCC-BY 4.0 International license.

Figure 2 - figure supplement 5. Mutational landscape of the METΔEx14 kinase domain under 11 ATP-competitive inhibitor selection. (A) Distributions of all variants (**wild-type** synonymous, early stop, and missense) for each condition, scored with Rosace and normalized to the growth rate of the DMSO control population. (B) Correlation plots for all mutational fitness scores for each drug against DMSO, fitted with a linear regression and Pearson's R value displayed. (C) Heatmap showing the Pearson's R correlation for each condition against each other, annotated by condition and inhibitor type. Correlations are colored according to a scale bar from gray to blue (low to high correlation).

bioRxiv preprint doi: https://doi.org/10.1101/2024.07.16.603579; this version posted December 5, 2024. The copyright holder for this preprint (which was not certified by peer review) is the author/funder, who has granted bioRxiv a license to display the preprint in perpetuity. It is made available under aCC-BY 4.0 International license.

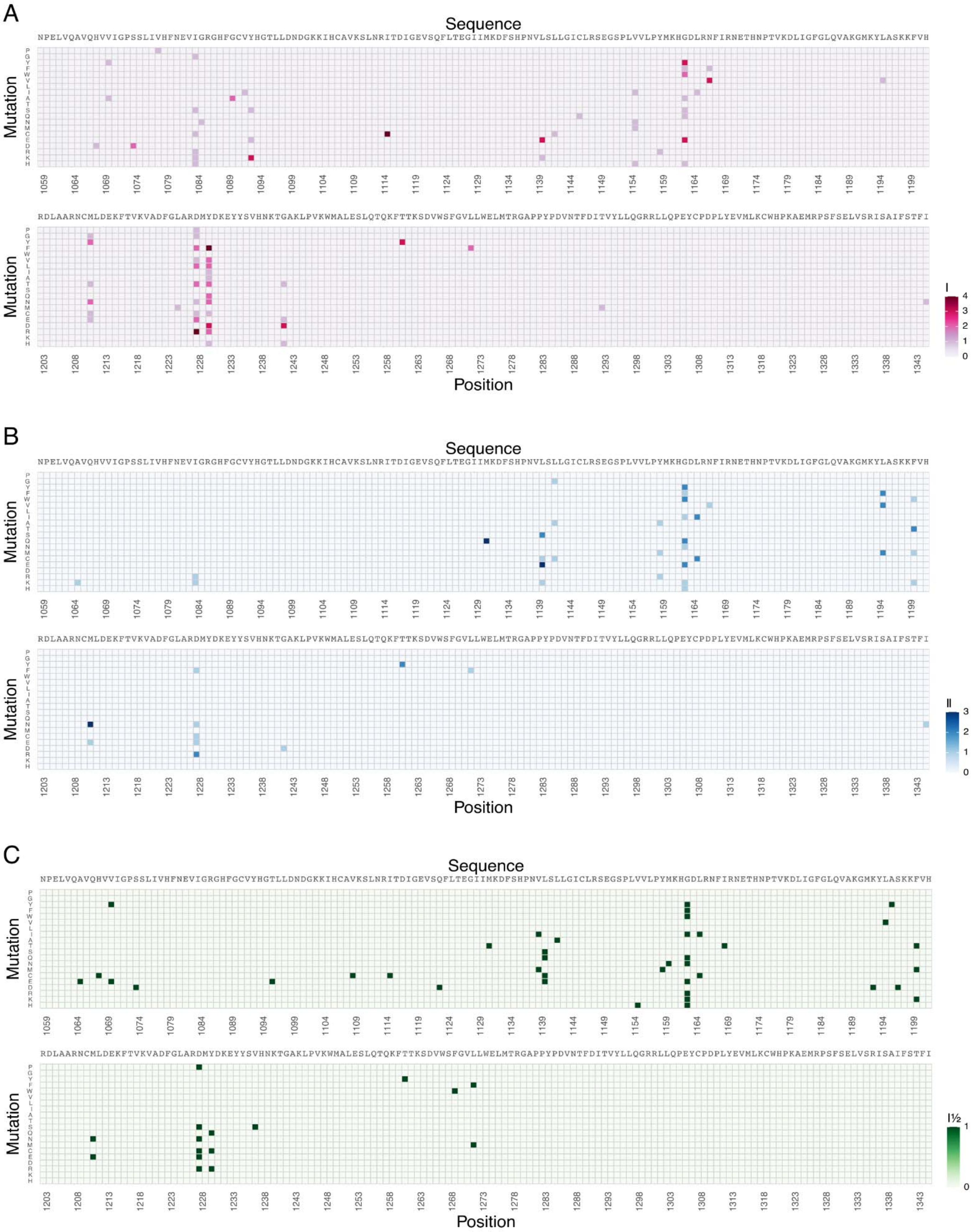

bioRxiv preprint doi: https://doi.org/10.1101/2024.07.16.603579; this version posted December 5, 2024. The copyright holder for this preprint (which was not certified by peer review) is the author/funder, who has granted bioRxiv a license to display the preprint in perpetuity. It is made available under aCC-BY 4.0 International license.

Figure 6 - figure supplement 1. Statistically filtered resistance mutations for grouped type I, type II, and type I½ inhibitors for MET. (A-C) Heatmaps of the sum of resistance mutations grouped for type I (pink), type II (blue), and type I½ (green) for MET.

bioRxiv preprint doi: https://doi.org/10.1101/2024.07.16.603579; this version posted December 5, 2024. The copyright holder for this preprint (which was not certified by peer review) is the author/funder, who has granted bioRxiv a license to display the preprint in perpetuity. It is made available under aCC-BY 4.0 International license.

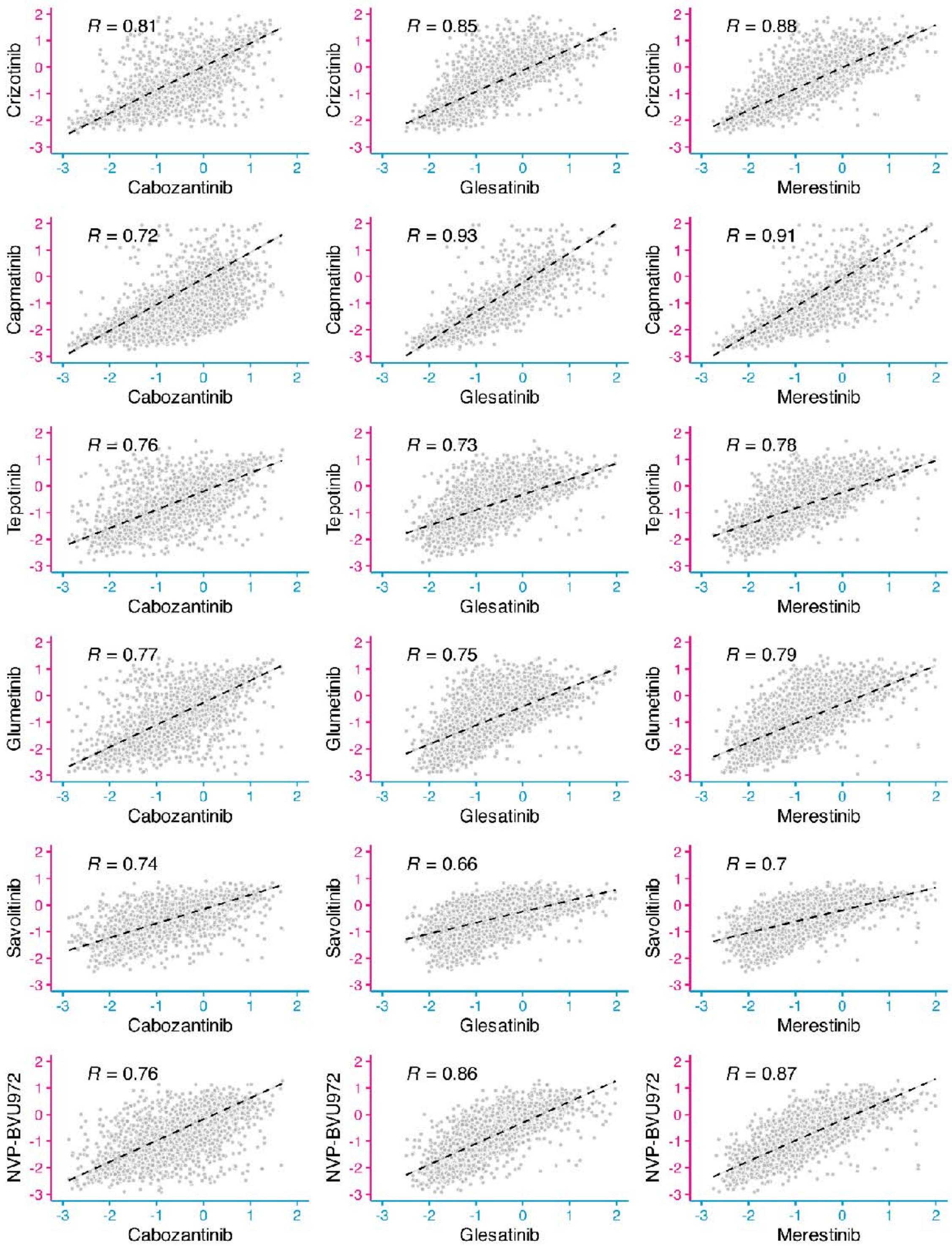

Figure 7 - figure supplement 1. Cross-comparison of type I and type II inhibitor pairs. Scatter plots of each type II inhibitor fitness scores (cabozantinib, glesatinib analog, merestinib; axis in blue) against each type I inhibitor (crizotinib, capmatinib, tepotinib, glumetinib, savolitinib, NVP-BVU972; axis in pink).

bioRxiv preprint doi: https://doi.org/10.1101/2024.07.16.603579; this version posted December 5, 2024. The copyright holder for this preprint (which was not certified by peer review) is the author/funder, who has granted bioRxiv a license to display the preprint in perpetuity. It is made available under aCC-BY 4.0 International license.

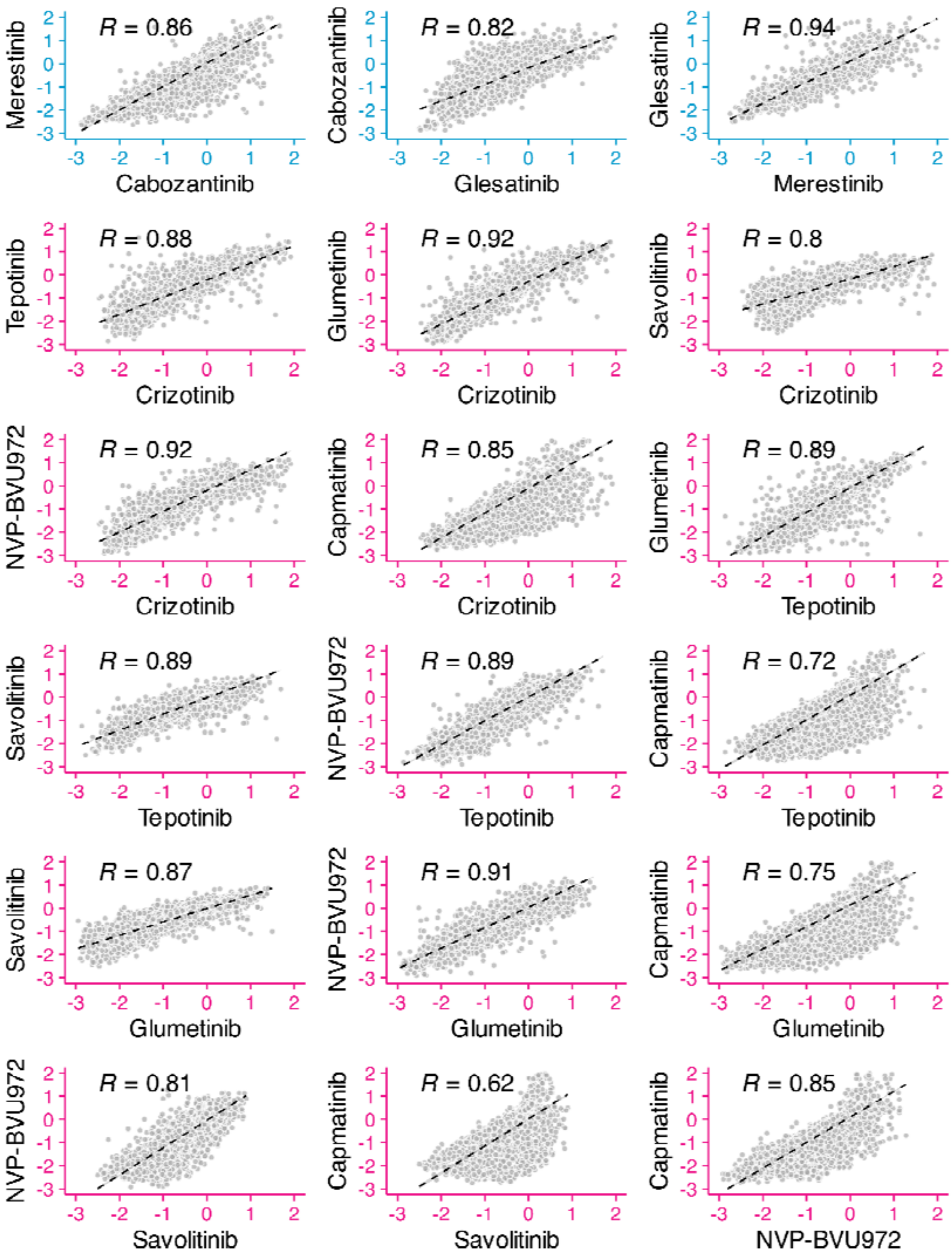

Figure 7 - figure supplement 2. Cross-comparison analysis of inhibitors within the same type. Scatter plots of each inhibitor pair within the type II group (cabozantinib, glesatinib analog, merestinib; axes in blue) and within the type I group (crizotinib, capmatinib, tepotinib, glumetinib, savolitinib, NVP-BVU972; axes in pink).

bioRxiv preprint doi: https://doi.org/10.1101/2024.07.16.603579; this version posted December 5, 2024. The copyright holder for this preprint (which was not certified by peer review) is the author/funder, who has granted bioRxiv a license to display the preprint in perpetuity. It is made available under aCC-BY 4.0 International license.

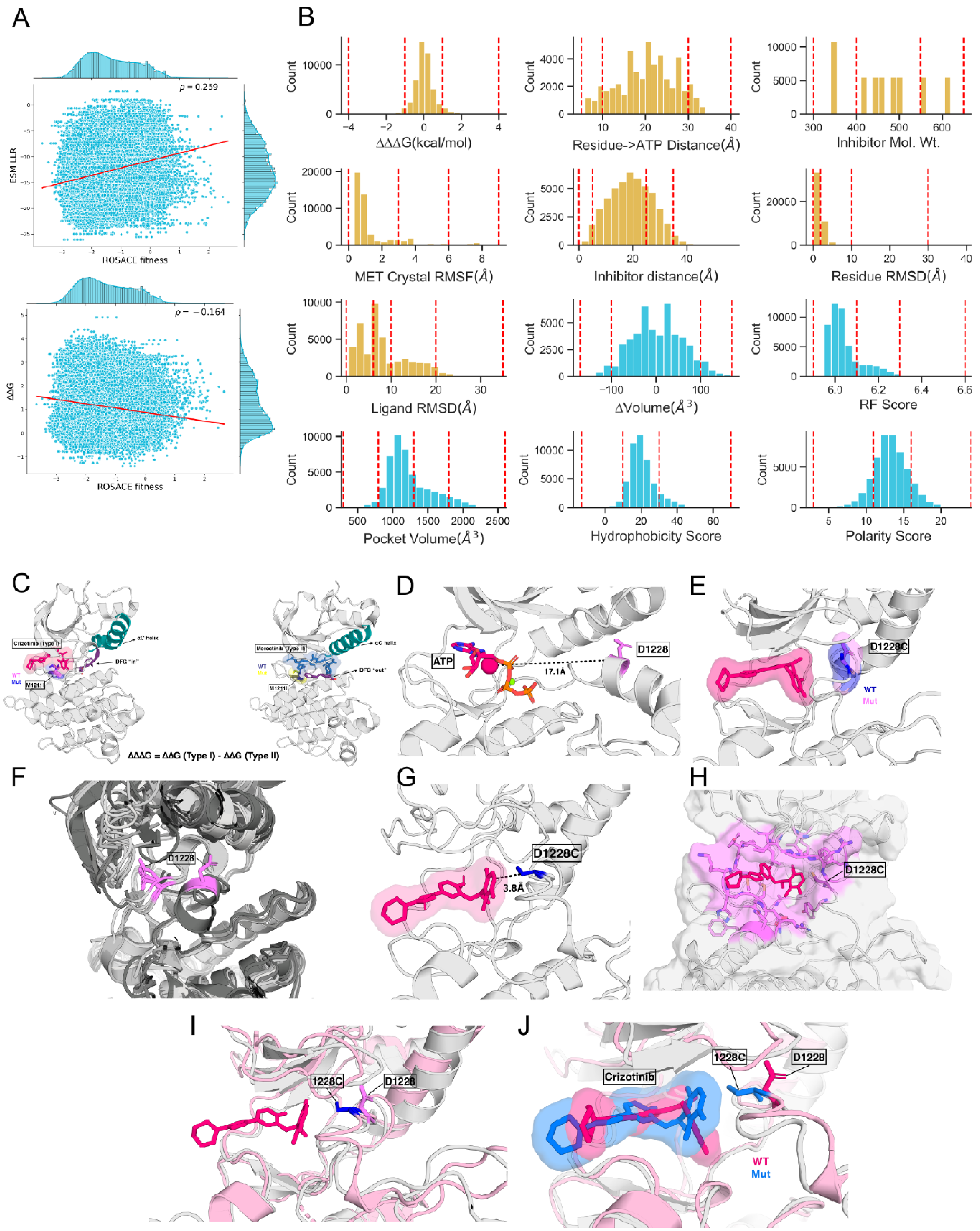

bioRxiv preprint doi: https://doi.org/10.1101/2024.07.16.603579; this version posted December 5, 2024. The copyright holder for this preprint (which was not certified by peer review) is the author/funder, who has granted bioRxiv a license to display the preprint in perpetuity. It is made available under aCC-BY 4.0 International license.

Figure 8 - figure supplement 1. Distribution and visualization of features used in the XGBoost machine learning models. (A) Distribution of ESM LLR vs. Experimental fitness (top) and ΔΔG vs. Experimental fitness (bottom). (B) Distribution of all features (except ESM LLR and ΔΔG) extracted and used for the XGBoost models. The features that were incorporated in the best performing model are shown in yellow. The red dashed lines within each distribution show the edges of bins used to bin the feature values. (C) ΔΔΔG calculated from predicted ΔΔG Type I (PDB 2WGJ) (left) and Type II (PDB 4EEV) (right) MET kinase structure by subtracting type II ΔΔG from type I ΔΔG. The key regions showing difference in conformation between type I and II structures are the DFG motif (purple) and aC helix (teal). (D) Calculation of "residue to ATP" distance feature for residue D1228 in ATP bound MET Kinase structure (3DKC) is shown. Centroid of the ATP molecule is shown as a pink sphere. (E) Example of ΔVolume feature calculation using the difference between the volume of Asp and Cys. (F) Ensemble of MET kinase domain crystal structures aligned and RMSF of a given residue (D1228 in this example). (G) The shortest distance between the inhibitor and a mutation calculated from the Umol predicted variant-inhibitor structure. (H) The binding pocket of crizotinib in the predicted Umol structure. Pocket volume, hydrophobicity score, polarity score and RF score are calculated from this binding site. (I) Residue RMSD feature is described by the Umol predicted structure of variant D1228C (pink) superposed onto the wild-type reference structure (PDB 2WGJ, gray) and RMSD between D1228 in the wild-type structure and 1228C in the variant structure. (J) Ligand RMSD feature The Umol predicted structure of variant D1228C (pink) superposed onto the wild-type reference structure (PDB 2WGJ, gray) and RMSD between crizotinib in the wild-type structure (pink) and in the variant structure (blue).
